# PP51 Pharmaceutical Innovativeness Index (PII): A New Framework For Assessing Health Technology Innovation Based On Therapeutic And Social Relevance

**DOI:** 10.1017/S0266462324002228

**Published:** 2025-01-07

**Authors:** Ludmila Gargano, Ariane André, Isabela Freitas, Francisco de Assis Acurcio, Juliana Alvares-Teodoro, Augusto Guerra

## Abstract

**Introduction:**

The value of a new pharmaceutical product should rely on its therapeutic benefits while also considering social health needs. Adapting health technology assessment (HTA) tools, we propose a transparent framework, with well-defined criteria and script, to determine the value of innovation (i.e., innovativeness) with clinical and methodological parameters, based on the social relevance and therapeutic value of new medicines.

**Methods:**

The study was developed by adapting HTA-based methods identified in the literature: the Italian Medicines Agency (AIFA) and the EValuation of pharmaceutical Innovations with regard to Therapeutic Advantage (EVITA) tool. A sample of oncology drugs approved by the U.S. Food and Drug Administration (FDA) between 2011 and 2021 were assessed by researchers trained in HTA tools using those methods. After assessing the sample, researchers discussed the results, difficulties, and issues experienced. Those issues were addressed, resulting in the creation of the new framework, which included the redefinition of domains, definition of classification criteria for each domain, and scores according to relevance.

**Results:**

The Pharmaceutical Innovativeness Index (PII) was proposed, with a script, domains, criteria, and an algorithm. The evaluation begins defining indication, outcomes, therapeutic alternatives, time perspective, and data sources. Four domains were considered: Therapeutic Need, which evaluates the existence and benefits of alternatives; Added Therapeutic Value, which talks about the incremental clinical benefit when compared to those alternatives (these first two domains were graded into five levels ranging from absent to maximum); Study Design and Methodological Quality, both classified into three levels. Classification criteria for each domain can be adapted according to the indication and relevant outcomes.

**Conclusions:**

The PII framework considers clinical and social value weighted by methodological limitation of available evidence to determine the value of innovation of pharmaceutical products. It stands out as a transparent, adaptable, and reproducible tool that aims to reduce the subjectivity of analyses and assist with decision-making. PII has the potential to inform decision-making processes involving value-based pricing, reimbursement, and research and development investment.

